# Improved ion detection sensitivity in mass spectrometry imaging using tapping-mode scanning probe electrospray ionization to visualize localized lipids in mouse testes

**DOI:** 10.1007/s00216-024-05641-x

**Published:** 2024-11-22

**Authors:** Yoichi Otsuka, Maki Okada, Tomomi Hashidate-Yoshida, Katsuyuki Nagata, Makoto Yamada, Motohito Goto, Mengze Sun, Hideo Shindou, Michisato Toyoda

**Affiliations:** 1 Department of Physics, Graduate School of Science, Osaka University, Toyonaka, Osaka Japan; 2 Forefront Research Center, Graduate School of Science, Osaka University, Toyonaka, Osaka Japan; 3 Department of Life Science, National Center for Global Health and Medicine, Shinjuku, Tokyo Japan; 4 Shimadzu Corporation, Nakagyo, Kyoto Japan; 5 Central Institute for Experimental Medicine and Life Science, Kawasaki, Kanagawa Japan; 6 Department of Medical Lipid Science, Graduate School of Medicine, The University of Tokyo, Bunkyo, Tokyo Japan

**Keywords:** Ambient sampling and ionization, Mass spectrometry imaging, Docosahexaenoic acid–containing phospholipids, Lipid localization, Mouse testis, Spermatogenesis

## Abstract

Mass spectrometry imaging (MSI) is a promising analytical method to visualize the distribution of lipids in biological tissues. To clarify the relationship between cellular distribution and lipid types in a tissue, it is crucial to achieve both an improvement in ion detection sensitivity and a reduction in the ionization area. We report methods for improving the efficiency of ion transfer to a mass spectrometer and miniaturizing the extraction area of a sample for tapping-mode scanning probe electrospray ionization (t-SPESI), atmospheric pressure sampling, and ionization methods. To verify the efficacy of the new t-SPESI measurement system, MSI was performed on mouse testes with a pixel size of 5 µm. Lipid images of the testes from wild-type (WT) and lysophospholipid acyltransferase 3 (LPLAT3) knockout mice revealed the characteristic distribution of docosahexaenoic acid–containing phospholipids (DHA-PLs). A comparison of the ion images obtained by MSI and optical images of the same tissues stained with hematoxylin and eosin suggested that the distribution of DHA-PLs was significantly altered by spermatogenesis in the WT mouse testes.

**Graphical abstract:**

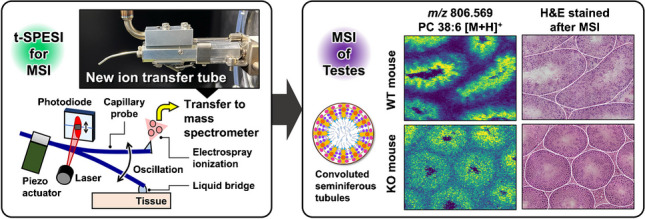

**Supplementary Information:**

The online version contains supplementary material available at 10.1007/s00216-024-05641-x.

## Introduction

In biological tissues, cells are functionally and structurally organized, and homeostasis is maintained through the high-level coordination of chemical reactions inside and outside the cells. The heterogeneity of the cellular network that constitutes the tissue leads to malignancy. Technology that visualizes the cellular transformation of diseased tissue based on molecular information is important for the advancement of pathological diagnosis, biomarker discovery, and elucidation of disease mechanisms.

Mass spectrometry imaging (MSI) is a technique used to visualize the distribution of tissue components. The components in the localized regions are ionized, and ionized molecules are introduced into a mass spectrometer to obtain mass spectra [1–3]. Information concerning the distribution of multiple components can be obtained from a single measurement by scanning the ionization region with respect to the sample surface. To visualize the distribution of components in tissues more clearly, methods for both the high-sensitivity measurement of ions and miniaturization of the ionization region are required to realize MSI with a smaller pixel size.

The development of ionization methods for MSI in microscopic regions has progressed. Ionization methods can generally be classified into three categories: methods that use ion beams, laser beams, and minute volumes of solvents. In secondary ion mass spectrometry (SIMS) [4], a sample is irradiated with a primary ion beam and the secondary ions emitted from the sample surface are measured. SIMS allows for nanoscale pixel sizes, but one issue is that the high-energy primary ion beam can cause fragmentation of biomolecules, limiting the information available in the mass spectrum. In matrix-assisted laser desorption ionization (MALDI) [5], the desorption and ionization of sample components are achieved by irradiating a focused laser beam onto a sample coated with an organic matrix on its surface. MSI with a pixel size of less than 1 µm has been achieved by reducing the laser spot diameter and using post-ionization technology [6]. The advantage of MALDI is that it can produce intact molecular ions, while selection of the matrix and coating method is necessary.

The ambient sampling and ionization (ASI) technique uses a small amount of solvent to perform liquid-phase extraction and electrospray ionization (ESI) of the components contained in a small area of the sample. This technique is characterized by its ability to perform MSI with minimal sample pretreatment. Desorption electrospray ionization (DESI) [7] uses a coaxial capillary to spray high-velocity charged droplets of solvent generated by ESI onto a sample. The charged droplets desorb locally and ionize the sample components. The extraction area was relatively large, at around 30–200 µm in diameter. Nanospray DESI [8] uses two capillary probes to supply a solvent with a high voltage to the sample for extraction and ESI. A feedback control technique using shear-force probes has been developed [9], and the MSI of mouse brain tissue with a pixel size of less than 7 µm has been reported [10].

**Fig. 1:**
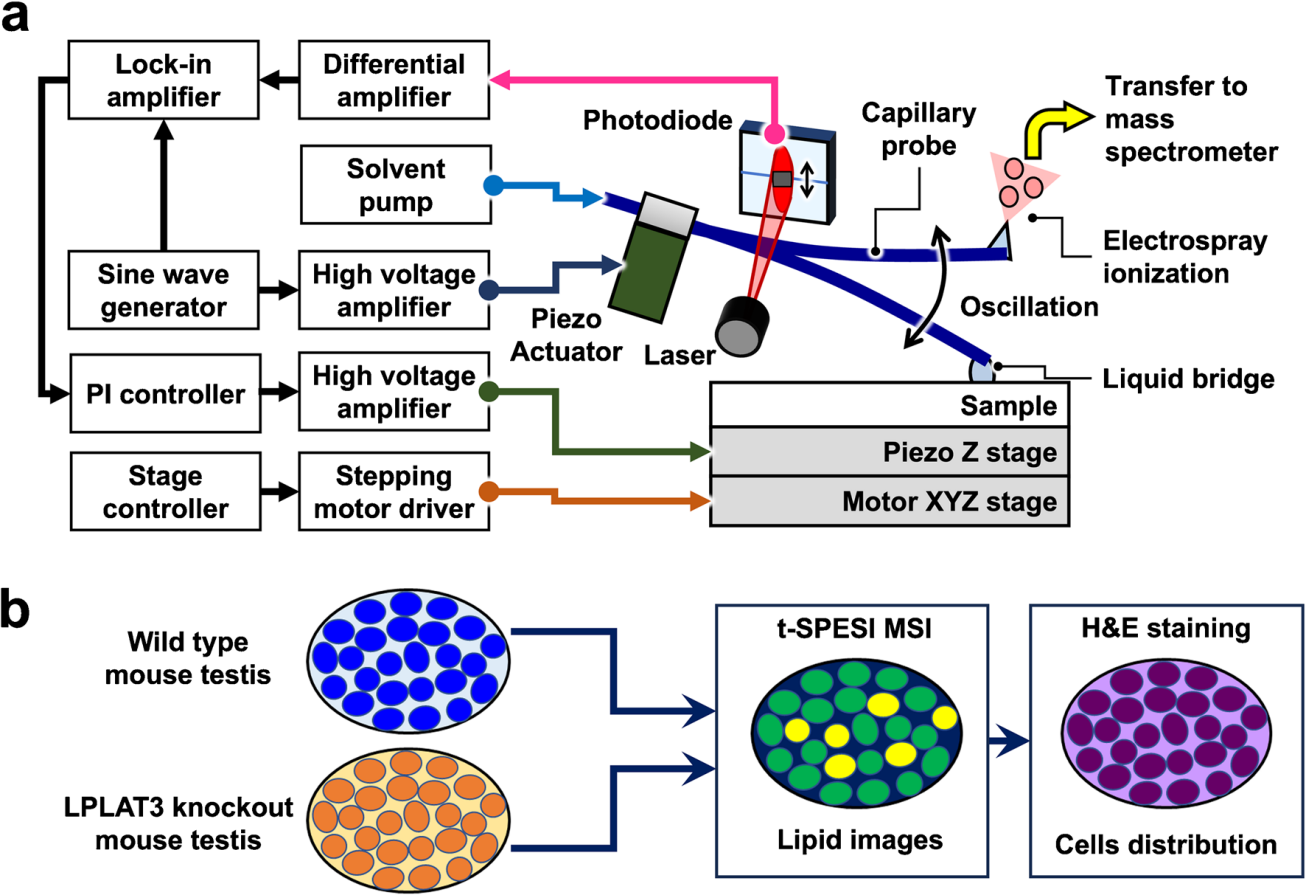
**a** Block diagram of the tapping-mode scanning probe electrospray ionization mass spectrometry imaging system. **b** Schematic showing the workflow of multimodal imaging of mouse testes

We previously reported tapping-mode scanning probe electrospray ionization (t-SPESI) [11], which uses an oscillating capillary probe for the local extraction and ionization of sample components in a small volume of solvent under atmospheric pressure. The distribution of lipids in tissues can be visualized without pretreatment. Figure 1a shows a schematic illustration of the t-SPESI. The probe was oscillated in the vertical direction using a piezoelectric actuator. The solvent flowing through the probe was charged by the application of high voltage. When the probe tip contacted the sample, a liquid bridge was formed and the sample components were extracted into the solvent. Then, the probe end was raised, and the extracted solution was mixed with the charged solvent flowing through the probe. Finally, the solution was subjected to ESI. The probe oscillation amplitude was monitored using a laser beam, photodiode, differential amplifier, and lock-in amplifier, and the height of the sample stage was continually adjusted by feedback control to maintain a constant amplitude to suppress the effects of the sample surface topography and stabilize the scanning [12]. To conduct MSI, the probe scanned the sample surface by repeating the above process, and the mass spectra of the ionized components were acquired.

In this study, we report an improvement in the signal intensity of ions by implementing a new ion transfer tube that is directly connected to a mass spectrometer. Moreover, to demonstrate that the new ion introduction method is effective for MSI of tissues, we examined the lipid distribution in mouse testes with a 5-µm pixel size. In addition to MSI, hematoxylin and eosin (H&E) staining was performed on the same tissues for optical microscopy (Fig. 1b). Comparisons between the two images revealed characteristic distributions of lipids inside the testes.

## Materials and methods

### t-SPESI measurement system

A t-SPESI system connected to a quadrupole time-of-flight mass spectrometer (LCMS-9030, Shimadzu, Japan) [13] was used. Capillary probe oscillation and sample position were controlled using in-house software coded in LabVIEW (version 2020; NI, USA). A new ion transfer tube was developed to introduce charged droplets and gas-phase ions generated from the tip of the capillary probe into the mass spectrometer with high efficiency. A nanoflow pump (LC-20ADnano, Shimadzu) was used to supply solvent through the capillary probe.

### Comparison of mass spectra obtained with indirect and direct ion transfer tubes

Sodium iodide solution (400 mg/L in pure water/MeOH = 1:1 v/v) was supplied to a silica emitter (FS360-20–10-N, New Objective, USA) with a 10-µm tip opening. A syringe pump (Legato 101, KD Scientific, USA) was used to set the flow rate to 200 nL/min. The silica emitter was attached to the probe holder, and the emitter tip was positioned at approximately 560 µm from the ion transfer tube. The voltages applied to the sample solution were 3 kV and − 3 kV for the positive and negative ion mode measurements, respectively. Mass spectra were measured every 20 ms for a total of 30 s, and the signal intensities of the cluster ions in averaged mass spectra were compared. In the indirect ion transfer method, the pressure in the chamber was 89.7 kPa, when the pumping rate of the dry pump connected to the chamber was 0.6 L/min.

### Capillary probes and solvent used for MSI

The solvent used was a mixture of *N*,*N*-dimethylformamide (LC grade; Nacalai Tesque, Japan) and methanol (LC grade; Nacalai Tesque). Capillary probes (approximately 4-µm aperture) were fabricated using a laser puller (P-2000, Sutter Instrument Co., USA) and fused silica tubes (30-µm inner diameter, 360-µm outer diameter; Molex, USA).

### Effect of ion transfer tube on the signal intensity of rhodamine B ions

We measured a red marker line (Ultra Fine Point, Red, 37,002, Sharpie, USA) on a glass substrate (Micro Slide Glass S1126; Matsunami Glass Ind. Ltd., Japan). The sample was prepared by drawing a single line. The intensities of the positive-ion peaks derived from rhodamine B [14] were compared. The voltage applied to the solvent was 4.0 kV. The solvent flow rate was 10 nL/min. The oscillation frequency of the probe was 625.6 Hz [15]. The area of the imaging was 120 µm × 1250 µm.

### Animals

*Agpat3* (lysophospholipid acyltransferase 3, LPLAT3) global knockout (KO) mice were provided by T. Sasaki, Institute of Science Tokyo. All mice were housed in an air-conditioned animal room at 23 ± 2 °C with a relative humidity of 40–60% under specific-pathogen-free (SPF) conditions, with a 12-h light and dark cycle (08:00–20:00/20:00–08:00), and had ad libitum access to water. *Agpat3* (LPLAT3) KO mice were generated as previously described [16]. The mice were euthanized at 19 weeks of age to collect testes. Mouse testes were collected from one wild-type (WT) and KO mouse.

### Testis sections for MSI

Frozen tissue sections were used for MSI. Tissue blocks were embedded in 2% sodium carboxymethyl cellulose (Wako, Japan) and sectioned at 10 µm with a Cryostat HM525NX (Thermo Scientific, USA); the sections were then mounted on a slide glass (Frost Slide Glass Edge Grinding No. 1, Matsunami Glass Ind., Ltd., Japan). The samples were placed in conical tubes containing silica gel and stored at − 80 °C. The tubes were thawed at room temperature prior to measurements, and the samples were used for MSI without preparation.

### MSI of mouse testes

The mass spectrum for a single pixel in an ion image was obtained every 50 ms in positive ion mode. The *m*/*z* range was 100–1200. The speed of the probe scan was 100 µm/s. These settings corresponded to a 5-µm pixel size in the ion image. A 550 µm × 1000 µm area was measured, in which 110 probe scanning lines over 1000 µm were performed at a 5-µm line spacing. The solvent flow rate was 5 nL/min. The voltage applied to the solvent was 4.0 kV. The oscillation frequencies of the probe were 625.6 Hz (WT mouse) and 607.1 Hz (KO mouse). To reduce background ion signals, clean air was supplied to the area close to the ion transfer tube via a Teflon tube connected to an active background-ion reduction device (ABIRD; ESI Source Solutions, USA).

### H&E staining of tissue sections

Tissue sections subjected to MSI were immersed in hematoxylin solution (Sigma-Aldrich, Germany) for 1 min and then immersed in ultrapure water for 1 min to remove excess hematoxylin. The samples were then immersed in 1% Eosin Y solution (Fujifilm, Japan) for 5 s, followed by three successive immersions in ultrapure water for 1 min. After immersion in ethanol (Nacalai Tesque) for 2 min for dehydration, the tissue was immersed in xylene (Nacalai Tesque) for 2 min to permeabilize the cells. After drying the tissue sections, a sealant (Excel mount 220, Falma, Japan) and cover glass (C024321, Matsunami) were placed on top and allowed to dry overnight. H&E-stained testis tissue was imaged using an inverted optical microscope (TE-2000U, Nikon, Japan) and a × 40 objective lens (Plan Fluor, ELWD, Ph2 DM, Nikon). Bright-field images were captured and tiled using NIS-Elements software (Nikon).

### Data analysis

Imaging data were analyzed using IMAGEREVEAL (version 1.21.0.11652, Shimadzu). Data preprocessing was performed by normalizing the mass spectrum at each pixel to the total ion current (TIC). The data were subjected to a targeted analysis. Previously, quantitative analysis of 18 types of phosphatidylcholine (PC) and phosphatidylethanolamine (PE) by liquid chromatography-mass spectrometry (LC–MS) has been reported [16]. In the present study, the ion peaks of these lipids were examined using MSI data. The *m*/*z* values of the lipid ions for protonated molecules ([M + H]^+^), sodium adduct molecules ([M + Na]^+^), and potassium adduct molecules ([M + K]^+^) were obtained from the LIPID MAPS database [17]. As a result, the ion peaks of the 16 PCs and 7 PEs were assigned (Supplementary Table 1). A list of the assigned lipids, including PC, ether-linked PC (PC O −), alkynyl ether–linked PC (PC P −), PE, ether-linked PE (PE O −), and alkynyl ether–linked PE (PE P −) is provided in Supplementary Table 2. Ion images were generated using the sum of the signal intensities in the *m*/*z* 0.005 range, centered on the *m*/*z* value for the lipid ions (Supplementary Fig. 1).

## Results and discussions

### Improvement of t-SPESI system

**Fig. 2:**
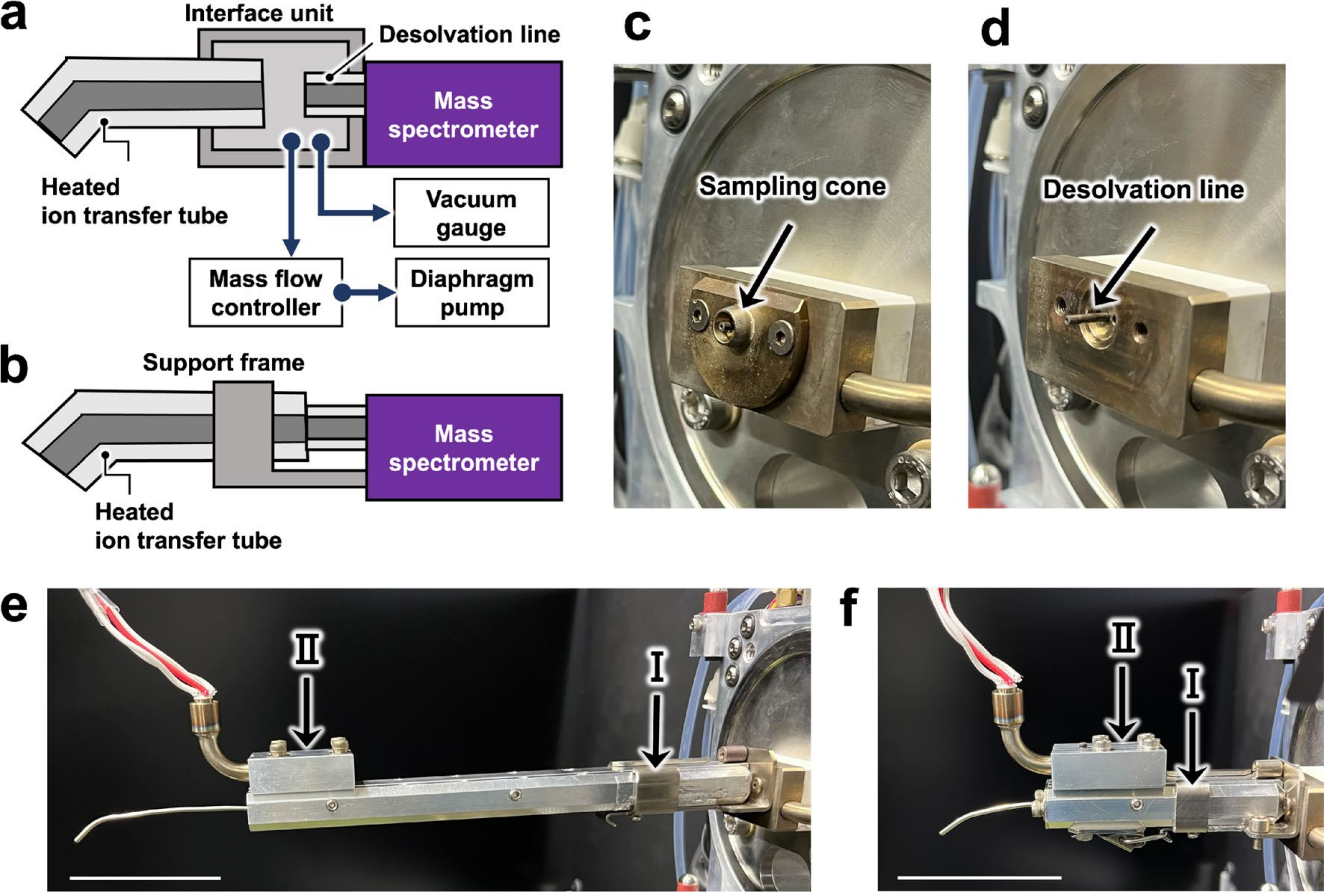
Schematic representation of the ion transfer tube used to guide ions generated by t-SPESI to the mass spectrometer. **a** Previous method. **b** New method. Photographs of the sampling cone (**c**) and the desolvation line (**d**) of the mass spectrometer, respectively. Side views of 24-cm (**e**) and 11-cm (**f**) ion transfer tubes connected directly to the desolvation line. The supporting frame, indicated by arrow I, was fixed to the block heater of the mass spectrometer, to which the ion transport tube was fixed. The ion transport tube was heated using an attached heater block (arrow II). Scale bars, 5 cm

We implemented a new direct connection of an ion transfer tube to enhance the signal intensity of ions generated by t-SPESI. In the previous system, a homemade interface unit was connected to the mass spectrometer (Fig. 2a). Inside the interface unit, the desolvation line (DL), which is the ion inlet of the mass spectrometer, and the ion transfer tube faced each other, with a 1-mm gap between them [13]. In this gap, the gas-phase ions produced in the atmosphere were assumed to be spatially spread in front of the DL to reduce their input to the mass spectrometer. In the new configuration, the ion transfer tube was directly connected to the DL (Fig. 2b). The sampling cone, which was used to flow nitrogen gas to dry the charged droplets generated by ESI, was removed to expose the DL (Fig. 2c, d). The support frame was fixed to the screw hole of the sampling cone, and a new ion transfer tube with an aluminum heating block was mounted on the frame. Two types of tubes with different lengths were tested (24 cm and 11 cm long, 1.8-mm outer diameter, 0.8-mm inner diameter) (Fig. 2e, f).

**Fig. 3:**
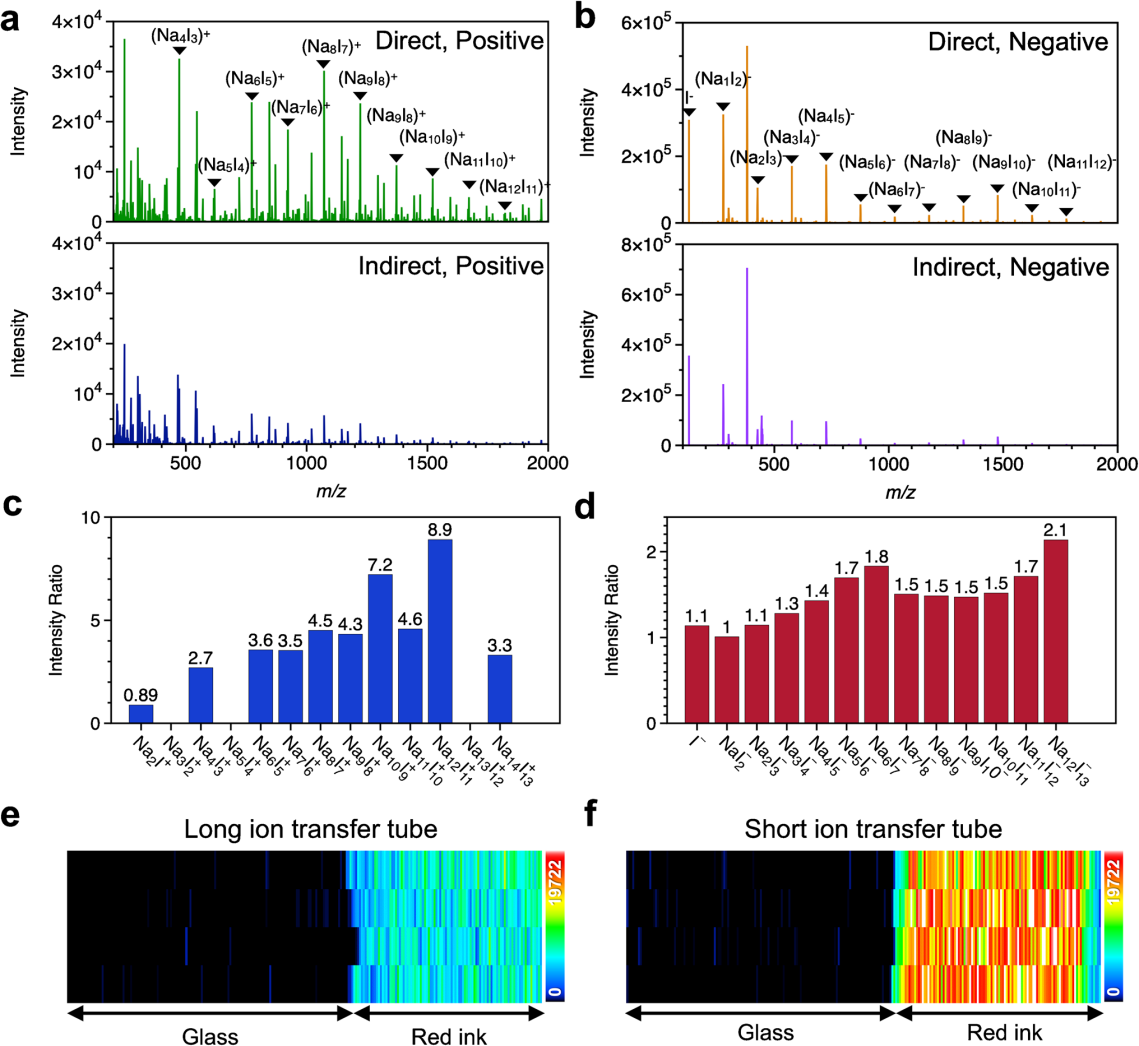
Comparison of mass spectra obtained with indirect and direct ion transfer tubes. Mass spectra of NaI cluster ions measured in positive (**a**) and negative (**b**) ion modes. The signal intensity ratios (*R*_i_) for the positive (**c**) and negative (**d**) ion modes, respectively. The ion images of the rhodamine B fragment obtained with 24-cm-long (**e**) and 11-cm-long (**f**) ion transfer tubes, respectively. The range of the signal intensity is given by a color scale

We compared the signal intensities of sodium iodide (NaI) cluster ions produced by ESI between previous and new ion introduction methods using a 24-cm-long tube. Figure 3a and b shows the mass spectra measured in the positive and negative ion modes, respectively. The signal intensities of the cluster ions were increased by the direct connection. Figure 3c and d shows the signal intensity ratios (*R*_i_) for the positive and negative ion modes, respectively. The signal intensity ratio (*R*_i_) of each cluster ion and its average (*R*_average_) were calculated as follows:

1 $${R}_{\mathrm{i}}={~}^{{I}_{\mathrm{direct}}}\!\left/ \!{~}_{{I}_{\mathrm{indirect}}}\right.$$

2 $${R}_{\mathrm{average}}=\frac{1}{n}\sum\nolimits_{i=1}^{n}{R}_{\mathrm{i}}$$

where *n* is the total number of cluster ions and *R*_i_ is the signal ratio of cluster ions. *I*_direct_ and *I*_indirect_ are the peak intensities of the cluster ions measured using the direct and indirect transfer methods, respectively. The averaged signal intensity ratio (*R*_average_) was 4.4 and 1.5 for the positive ion and negative ion modes, respectively. Hence, there was an improvement in the ion transfer efficiency into the mass spectrometer via the direct connection of the ion transfer tube to the DL.

To evaluate the effect of tube length on the ion signal intensity of MSI, ionization of a rhodamine B pattern on a glass substrate by t-SPESI was performed with 24-cm and 11-cm ion transfer tubes. Figure 3e and f shows ion images of *m*/*z* 399.171 (C_3_H_8_ loss of rhodamine B) obtained with long and short ion transfer tubes, respectively. The range of the signal intensity is given by a color scale. The average intensity in the rhodamine B pattern increased 2.6 times with the short ion transfer tube. By coupling the short tube directly to the DL, we were able to enhance the detection sensitivity of the positive ion mode by a factor of 10 compared to the previous ion transport tube, assuming that the ion transfer efficiency remained constant regardless of the type of ionized molecule. In addition to the implementation of the new ion transfer tube, we also improved the solvent supplement for extraction. To minimize the extraction area, it is necessary to have a small probe tip and stably supply a small volume of solvent. Thus, we utilized homemade probes with a 4-µm aperture which was fabricated with a laser puller. Moreover, a nanoflow pump was used instead of a syringe pump to ensure stable solvent flow.

### Comparison of lipid ion images between WT and KO mouse testes

**Fig. 4:**
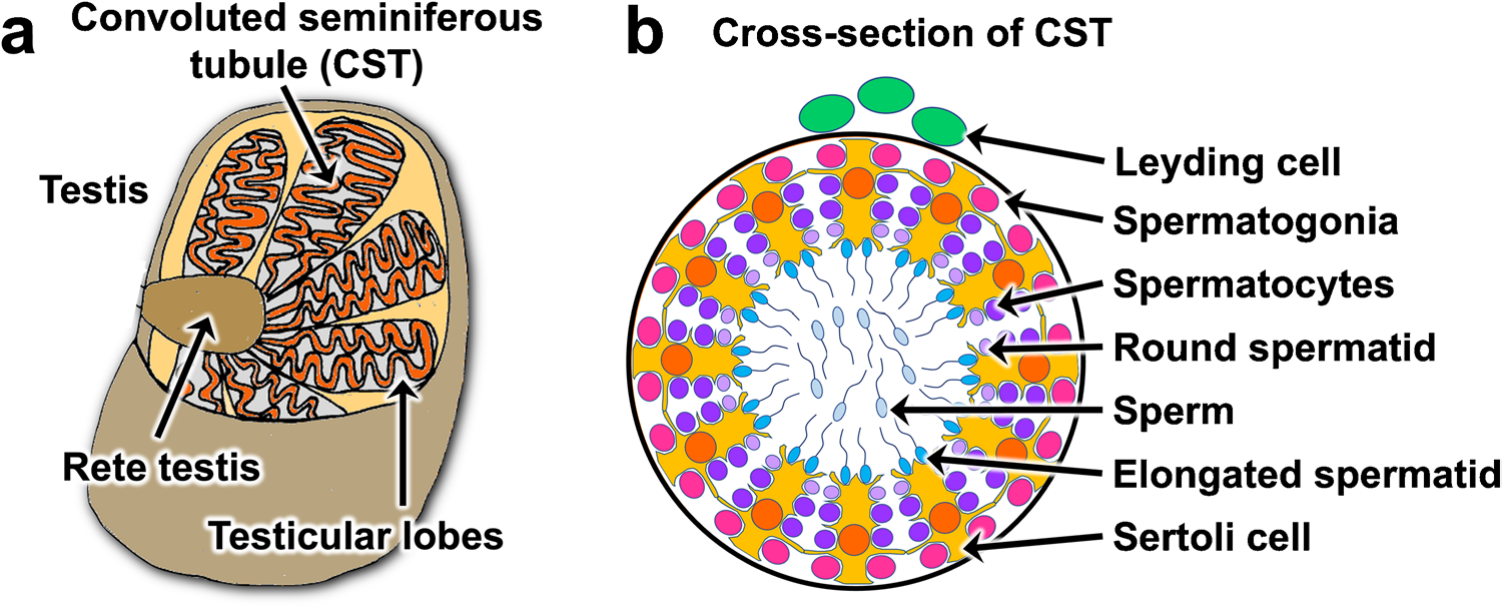
**a** Schematic representation of testicular tissue. **b** Schematic representation of cells in the cross-section of the convoluted seminiferous tubule

To verify the usefulness of the new t-SPESI measurement system, MSI was conducted on mouse testes and the distribution of lipids was visualized with a pixel size of 5 µm. Also, hematoxylin and eosin (H&E) staining was performed on the tissues used for MSI to compare the lipid ion images with the optical images. Sperms are produced in the convoluted seminiferous tubules (CSTs) in the testes, as shown schematically in Fig. 4a. A high density of CSTs is contained inside the testicular lobes, which are complex tubular structures with approximately 200-µm diameters that form a loop, leading to a rete testis [18]. Figure 4b shows a schematic cross-section of the CST. From the basal side toward the lumen, spermatogonia, spermatocytes, round spermatids, and elongated spermatids are arranged [19]. Sertoli cells supply nutrients to sperm cells [20] and remove their cytoplasm during the formation of the sperm head. Spermatogenesis in mice has 12 stages, according to the shape of the sperm cell and the stage of nuclear development. Because differentiation occurs along the CST, the spermatogenic area migrates along the CST in a wave-like manner. Therefore, four layers of differentiated germ cells at different stages were observed in CST cross-sections [21].

Glycerophospholipids such as docosahexaenoic acid–containing phospholipids (DHA-PLs) are one of the main components of the cell membrane. DHA-PLs contain at least one DHA, which has one acyl chain with 22 carbons and 6 double bonds. The expression of LPLAT3 (or LPAAT3/AGPAT3) is induced in the mouse testis, retina, or other tissues depending on age and is important to produce DHA-PLs [16, 22–24]. LC–MS revealed decreased DHA-PLs in the testes of LPLAT3 KO mice [16]. In WT mice, flexible cell membranes enriched with DHA-PLs are present in the CST, allowing for lysosomal degradation via endocytosis through tubulobulbar complexes and normal sperm formation. In contrast, deficiency of DHA-PLs in KO mice suppresses the removal of sperm cell cytoplasm by Sertoli cells, resulting in abnormal head morphology, which reduces sperm fertilization capability [16]. Spatial and chemical changes in the cells would be induced in the CST during spermatogenesis. Therefore, it is important to visualize the distribution of lipids in the testes to understand the lipids involved in spermatogenesis.

**Fig. 5:**
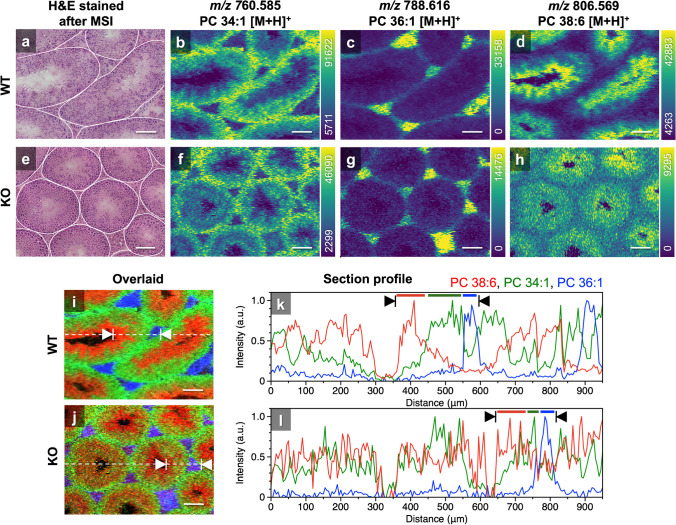
Comparison of optical microscopy images and lipid ion images. The relative signal intensity is indicated by color bars. **a** H&E-stained WT testis. **b**–**d** Ion images of WT testis. **e** H&E-stained KO testis. **f**–**h** Ion images of KO testis. Overlaid images of WT (**i**) and KO (**j**) testes, respectively. **k**, **l** Section profiles of overlaid images. The dashed lines in the overlaid images indicate the positions of the profiles. Scale bars, 100 µm

Figure 5 shows a comparison of H&E-stained tissue and ion images of three representative lipids in the WT and KO testes. Figure 5a shows an optical microscopy image of H&E-stained WT testes that were used for MSI. White lines were added to visualize the CST boundaries. The cell shapes were maintained in the H&E-stained tissue; therefore, extraction and ionization by t-SPESI did not deform or etch the tissue sections. The three ion images of the WT testes showed characteristic distribution patterns localized in the CST and stroma, as shown in Fig. 5b–d. The ions with *m*/*z* 760.585, 788.616, and 806.569 were assigned to PC 34:1 [M + H]^+^, PC 36:1 [M + H]^+^, and PC 38:6 [M + H]^+^, respectively. In the PC 34:1 [M + H]^+^ image (Fig. 5b), the intensity increased in the CST outer regions relative to that in the inner regions, indicating that PC 34:1 was distributed more in the regions corresponding to spermatogonia and spermatocytes. The PC 36:1 [M + H]^+^ image (Fig. 5c) showed localization of the signal in the stromal region (blood vessels, nerves, and Leydig cells) outside the CSTs. In the PC 38:6 [M + H]^+^ (DHA-PL) image (Fig. 5d), the intensity increased inside the CST. Previously, LC–MS analysis of PC 38:6 revealed that PC 18:2_20:4 and PC 16:0_22:6 coexisted [16], with PC 16:0_22:6 being the major component. Therefore, the ion images of PC 38:6 [M + H]^+^ discussed in this study would primarily show the distribution of PC 16:0_22:6. Figure 5e shows an optical microscopy image of H&E-stained KO testis. Three ion images are shown in Fig. 5f–h. The PC 34:1 [M + H]^+^ and PC 36:1 [M + H]^+^ images were similar to those of WT testes, but their signal intensities were lower. In contrast, the intensity of PC 38:6 [M + H]^+^ inside the CST was much lower than that in the WT testes.

The overlaid images from Fig. 5b–d and f–h are shown in Fig. 5i and j, respectively, where the color scale of the signal intensity for PC 34:1 [M + H]^+^ is green, that for PC 36:1 [M + H]^+^ is blue, and that for PC 38:6 [M + H]^+^ is red. The results of the cross-sectional analysis of the overlaid images are shown in Fig. 5k and l. The lines corresponding to the cross-sections are indicated by dashed lines in the overlaid image. Ion intensities were normalized to compare relative changes in signal intensity. The ranges indicated by the arrows include the CST outer regions, CST inner regions, and stromal regions. In WT testes, the distribution of lipid ion intensity was clearly apparent in each region, whereas in KO testes, the distribution of lipid ions was not clearly separated, particularly in the interior of the CST. MSI data from five and three regions of WT and KO testes were examined, and similar results were obtained for CSTs in different locations.

### Comparison of magnified lipid ion images with H&E images

**Fig. 6:**
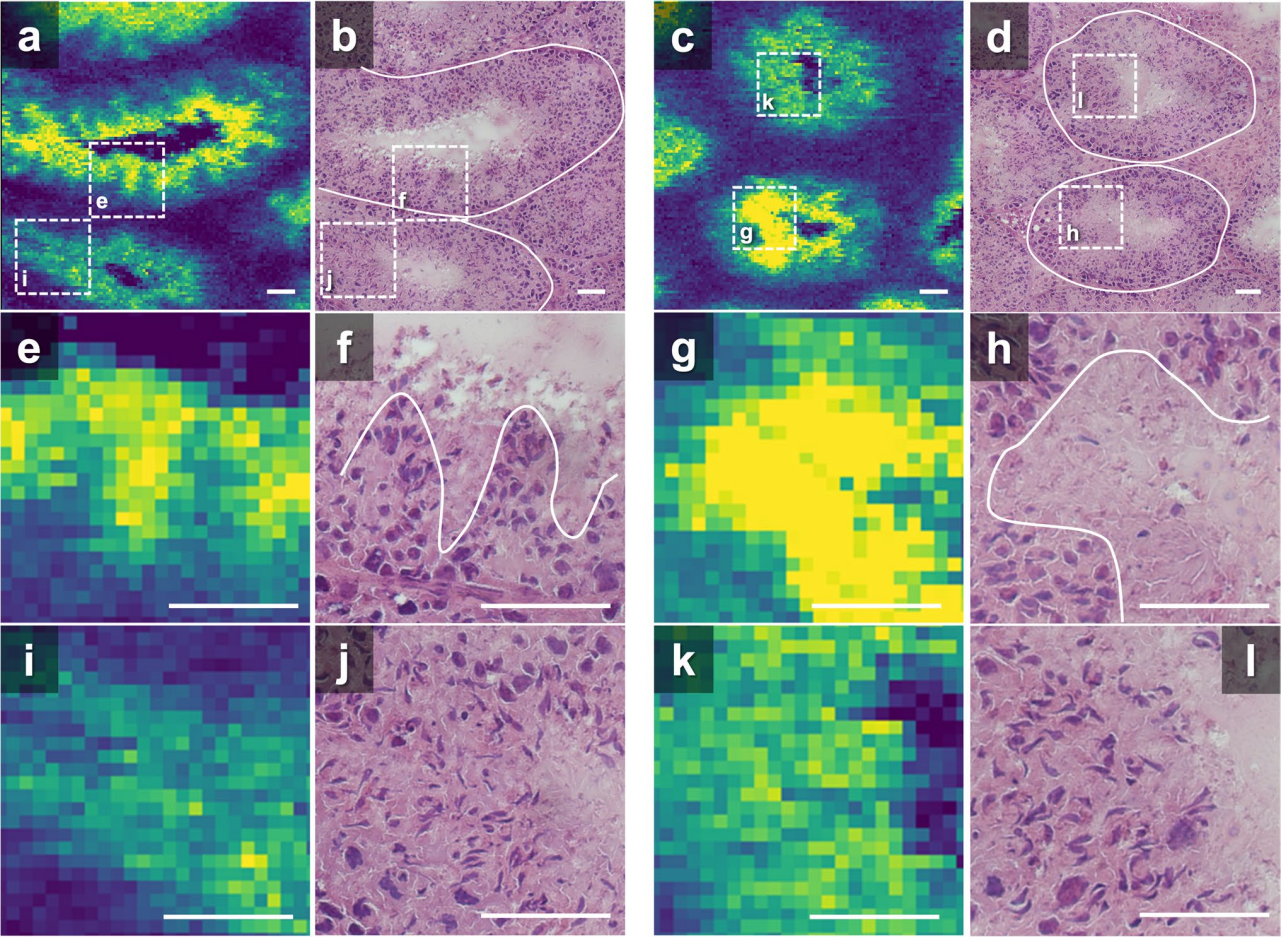
Comparison of magnified optical microscopy images and lipid ion images (*m*/*z* 806.569) of WT mouse. **a**, **c** Ion images. **b**, **d** Images of H&E-stained testes. **e**, **g**, **i**, **k** Magnified ion images. The corresponding regions are shown in **a** and **c**. **f**, **h**, **j**, **l** Magnified images of H&E-stained testes. Magnified regions are shown in **b** and **d**, respectively. Scale bars, 50 µm

The localization of DHA-PL inside the CST is discussed in terms of the relationship between sperm maturation and ion images. Images of PC 38:6 [M + H]^+^ and H&E-stained tissues from the WT testis are shown in Fig. 6a–d. Differences in intensity were observed for each CST. The central CST in Fig. 6a exhibited higher intensities than the lower CST, and the intensity is heterogeneous in the central CST. Similarly, the lower CST in Fig. 6c exhibited higher intensities than the upper CST. Enlarged images of the confined regions are shown in Fig. 6e–l; they are represented by dashed squares in Fig. 6a–d. The high-intensity region in Fig. 6e was similar to the region with few cell nuclei in the H&E image (Fig. 6f, the luminal side above the auxiliary line). An auxiliary line was added to Fig. 6f to visualize the border between different cell densities. This region might indicate areas where Sertoli cell cytoplasm was present. Sertoli cells are large somatic cells in the testes that are irregularly columnar in shape [19]. In contrast, ion intensity decreased in the region with a higher density of cell nuclei (Fig. 6f, below the auxiliary line). This clear localization in the ion image was also observed in other CSTs (Fig. 6g, h). In contrast, in another CST adjacent to the CST with high intensity, the intensity distribution was relatively homogeneous (Fig. 6i–l). From the cell morphologies in the H&E-stained tissues, the regions in Fig. 6f, h, j, and l were in stages IX–XI, IX–XI, VII–VIII, and XI–XII of spermatogenesis, respectively. The present results suggested that the intensity of PC 38:6 [M + H]^+^ within the CST increased in the region presumed to have Sertoli cell cytoplasm in the early phase of mouse sperm maturation (Fig. 6e, g). In contrast, the intensity was homogeneous during the late phase (Fig. 6i, k).

**Fig. 7:**
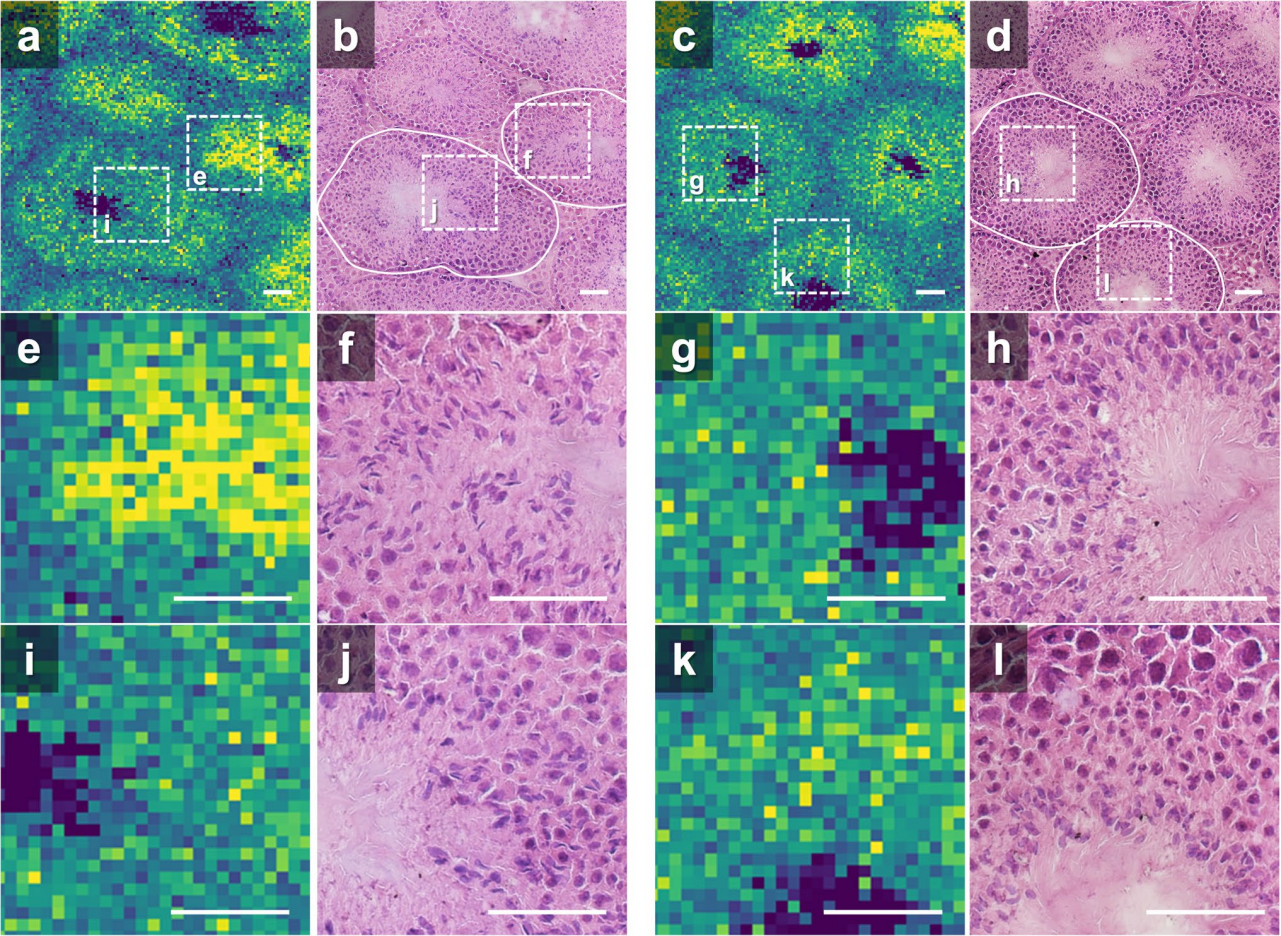
Comparison of magnified optical microscopy images and lipid ion images (*m*/*z* 806.569) of KO mouse. **a**, **c** Ion images. **b**, **d** Images of H&E-stained testes. **e**, **g**, **i**, **k** Magnified ion images. The corresponding regions are shown in **a** and **c**. **f**, **h**, **j**, **l** Magnified images of H&E-stained testes. Magnified regions are shown in **b** and **d**, respectively. Scale bars, 50 µm

Figure 7a–d shows a comparison of PC 38:6 [M + H]^+^ images of KO testes and optical images of H&E-stained testes. Magnified images of the restricted CST region are shown in Fig. 7e–l. The enlarged regions are indicated by dashed squares in Fig. 7a–d. The nuclei of sperm cells were observed in H&E-stained images. From the cell morphologies in the H&E-stained images, the regions in Fig. 7f, h, j, and l were considered to be in stages VIII, IX–XI, VIII–X, and IX–XI of spermatogenesis, respectively. As shown in Fig. 7e, there was a heterogeneous distribution of ion intensity within the CST, and the ion intensity increased in the region where the nucleus of the sperm head was present in the H&E-stained images (Fig. 7f). In contrast, ion images with different intensity distributions were observed in the CSTs adjacent to this CST and in other regions. The intensity was generally low and uniform in the region where spermatid nuclei were observed (Fig. 7g–l). These results suggested that sperm differentiation occurred in the CSTs of KO testes, but the intensity of PC 38:6 [M + H]^+^ was reduced and showed weak localization compared with that in the WT testes.

The flexibility of DHA-PLs enhances the ability of cell membranes to deform and form vesicles, and promotes rapid endocytosis [25]. DHA-PLs are also involved in the function and trafficking of various proteins required for proper sperm release and metabolic precursors of DHA-derived lipid mediators [26]. In this study, we compared lipid ion images and H&E-stained images of WT and KO testes, revealing differences in the localization of PC 38:6 in regions of putative Sertoli cells inside the CST.

MSI of the testes using MALDI has been reported previously. MSI of mouse testes from different age groups with a 25-µm pixel size showed that the lipid location and signal intensity varied with age [27]. The localization of DHA-PL in acyl-CoA synthetase isoform 6–deficient mouse testis (40-µm pixel size) [28] and lipid distributions in rat testes (10-µm pixel size) [29] were also reported. MSI of mouse testes using DESI with a pixel size of 25 µm was reported [30]. Our MSI results showed the distribution of localized lipids in the testes of WT and LPLAT3 KO mice, using a smaller pixel size.

MSI plays a complementary role to LC–MS, allowing direct visualization of the chemical state of tissue cellular networks. To discuss the biological relevance of lipid distribution information in detail, the acquisition of MSI data combined with tandem mass spectrometry (MS/MS) and the statistical analysis of biological tissue data acquired from several different biological replicates are important. Currently, we are working on techniques to stabilize measurements over extended periods to investigate multiple tissue sections.

## Conclusion

We introduced methods for the t-SPESI measurement system to improve the efficiency of ion transfer to the mass spectrometer and to miniaturize the extraction area of the sample components. For proof of concept, MSI was conducted on mouse testes at a pixel size of 5 µm. The ion images revealed distinct and characteristic distributions of DHA-PLs in the CSTs of the WT mouse testis. In contrast, their intensity and localization decreased in the CSTs of the LPLAT3 KO mouse testis. By comparing the ion images and H&E-stained testis images, we found that the localized distribution of DHA-PLs in the CSTs of WT mouse testis would be varied in response to sperm maturation. As technological advancements in MSI continue to evolve, we expect significant improvements in the quality and quantity of multidimensional chemical distribution information in biological tissues.

## Supplementary Information

Below is the link to the electronic supplementary material.

Supplementary file1 (DOCX 5.71 MB)

## Data Availability

The datasets generated and/or analyzed during the current study are available from the corresponding author upon reasonable request.
