# Supplementary material for: Improved ion detection sensitivity in mass spectrometry imaging using tapping-mode scanning probe electrospray ionization to visualize localized lipids in mouse testes
Source: Anal Bioanal Chem. 2024 Nov 22;417(2):275–86. doi: 10.1007/s00216-024-05641-x (PMC11698803; doi:10.1007/s00216-024-05641-x)
Supplement: Supplementary file 1 — Supplementary file1 (DOCX 5.71 MB) [file 216_2024_5641_MOESM1_ESM.docx]

**Supplementary Information**

**Improved ion detection sensitivity in mass spectrometry imaging using
tapping-mode scanning probe electrospray ionization to visualize localized lipids in mouse testes**

Yoichi Otsuka^a, b†^, Maki Okada^a^, Tomomi Hashidate-Yoshida^c^, Katsuyuki Nagata^c^, Makoto Yamada^d^, Motohito Goto^e^, Mengze Sun^a^, Hideo Shindou^c, f^, Michisato Toyoda^a,b^

*^a^ Department of Physics, Graduate School of Science, Osaka University, Japan.*

*^b^ Forefront Research Center, Graduate School of Science, Osaka University, Japan.*

*^c^ Department of Life Science, National Center for Global Health and Medicine, Japan*

*^d^ Shimadzu Corporation, Japan*

*^e^ Central Institute for Experimental Medicine and Life Science, Japan*

*^f^ Departments of Medical Lipid Science, Graduate School of Medicine, The University of Tokyo, Japan*

^†^Corresponding author

^†^Email: otsuka@phys.sci.osaka-u.ac.jp

[Table S1. List of lipids by LC/MS and the detected lipids by t-SPESI. 2](#_Toc180444731)

[Table S2. List of lipids used for analysis. 3](#_Toc180444732)

[Fig. S1. Comparison of ion images. 4](#_Toc180444733)

[References 7](#_Toc180444734)

# Table S1. List of lipids by LC/MS and the detected lipids by t-SPESI.

zs

The results by LC/MS were obtained from a previous study [1]. PC: phosphatidylcholine, PC O-: Ether linked PC, PC P-: alkynyl ether linked PC, PE: phosphatidylethanolamine, PE O-: Ether linked PE, PE P-: alkynyl ether linked PE. Circles indicate lipids for which ion peaks were detected in the mass spectra obtained using t-SPESI.

# Table S2. List of lipids used for analysis.

PC: phosphatidylcholine, PC O-: Ether linked PC, PC P-: alkynyl ether linked PC, PE: phosphatidylethanolamine, PE O-: Ether linked PE, PE P-: alkynyl ether linked PE, N.D.: Not detected.

# Fig. S1. Comparison of ion images.


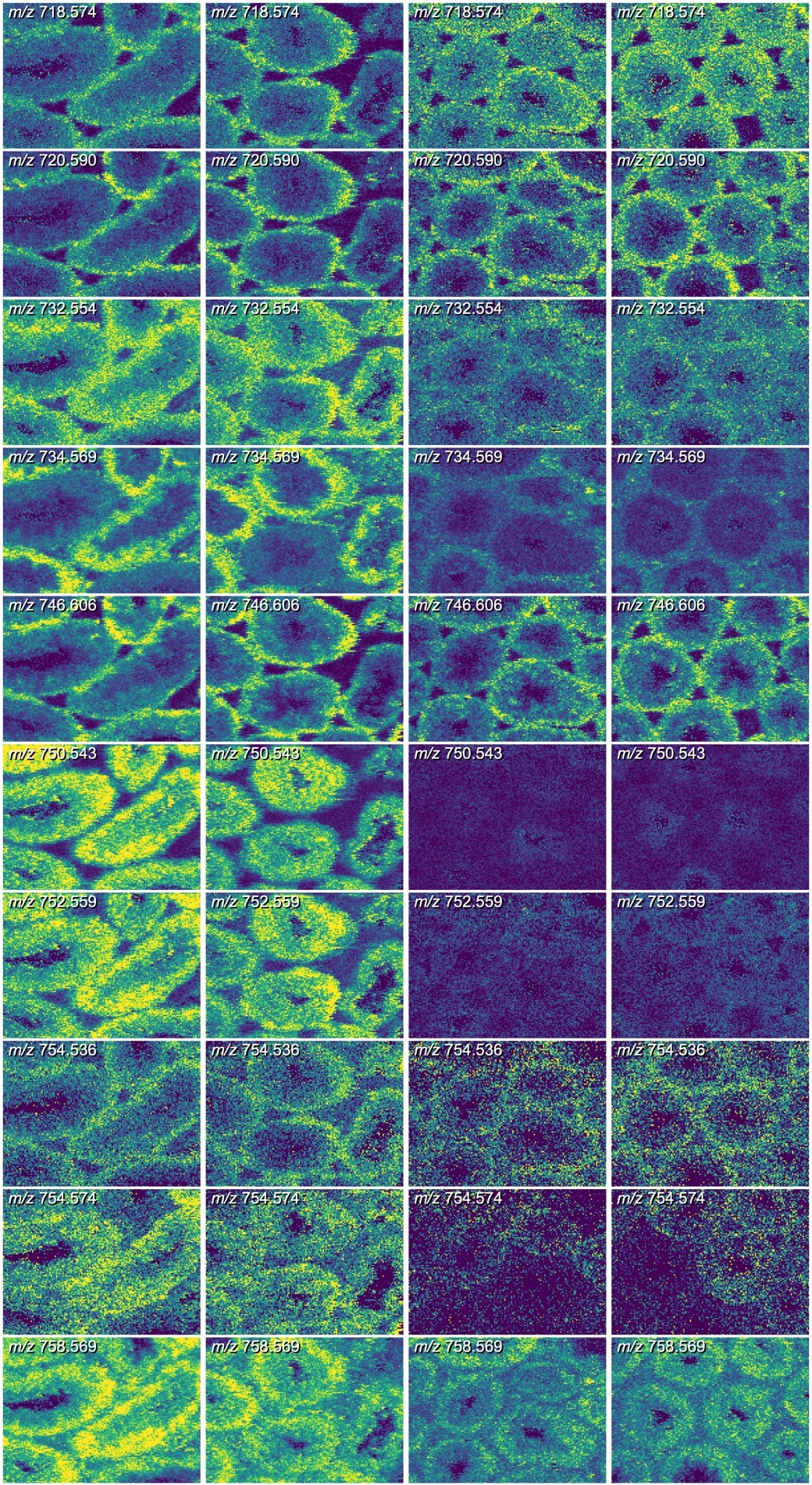


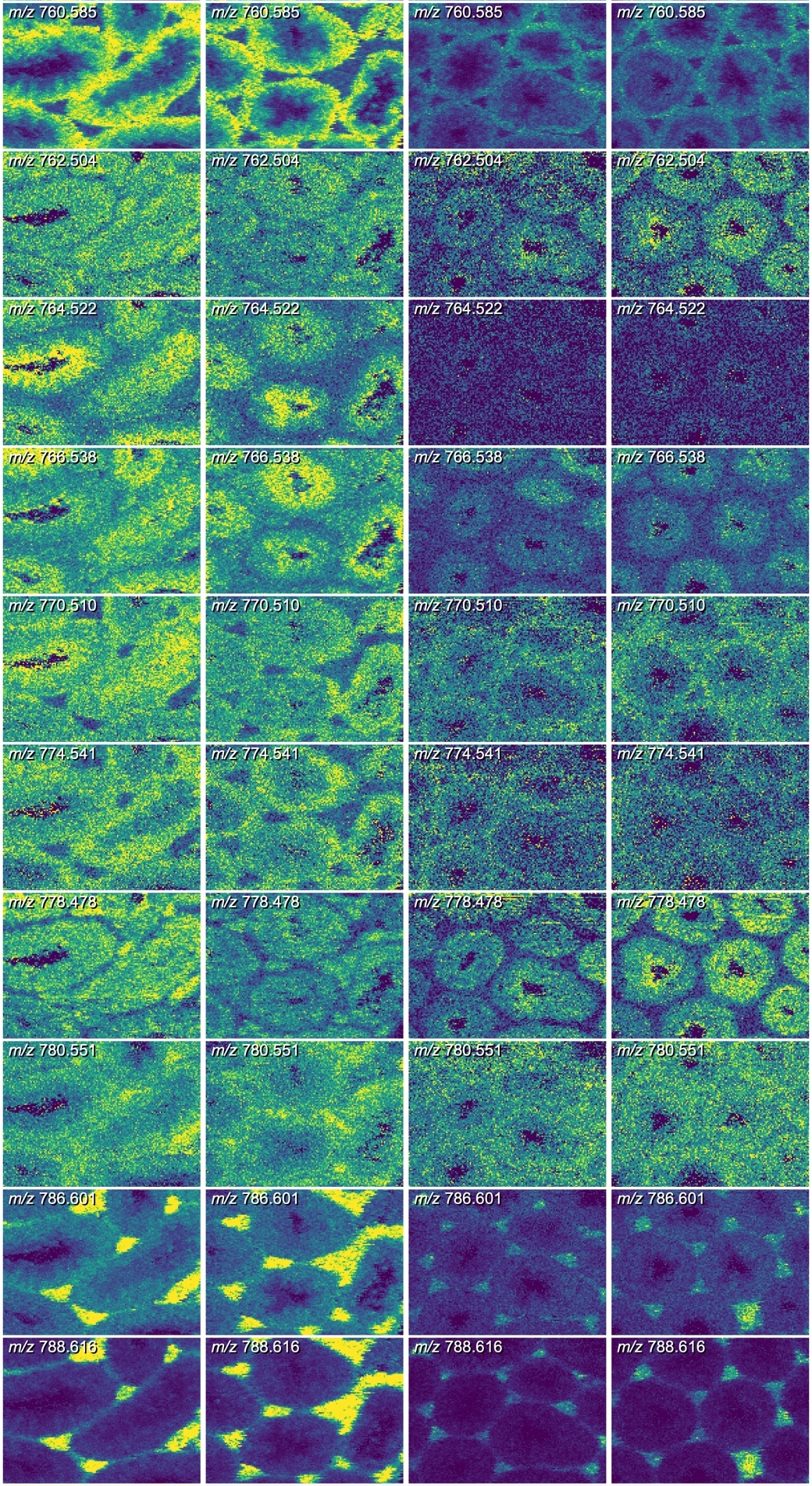


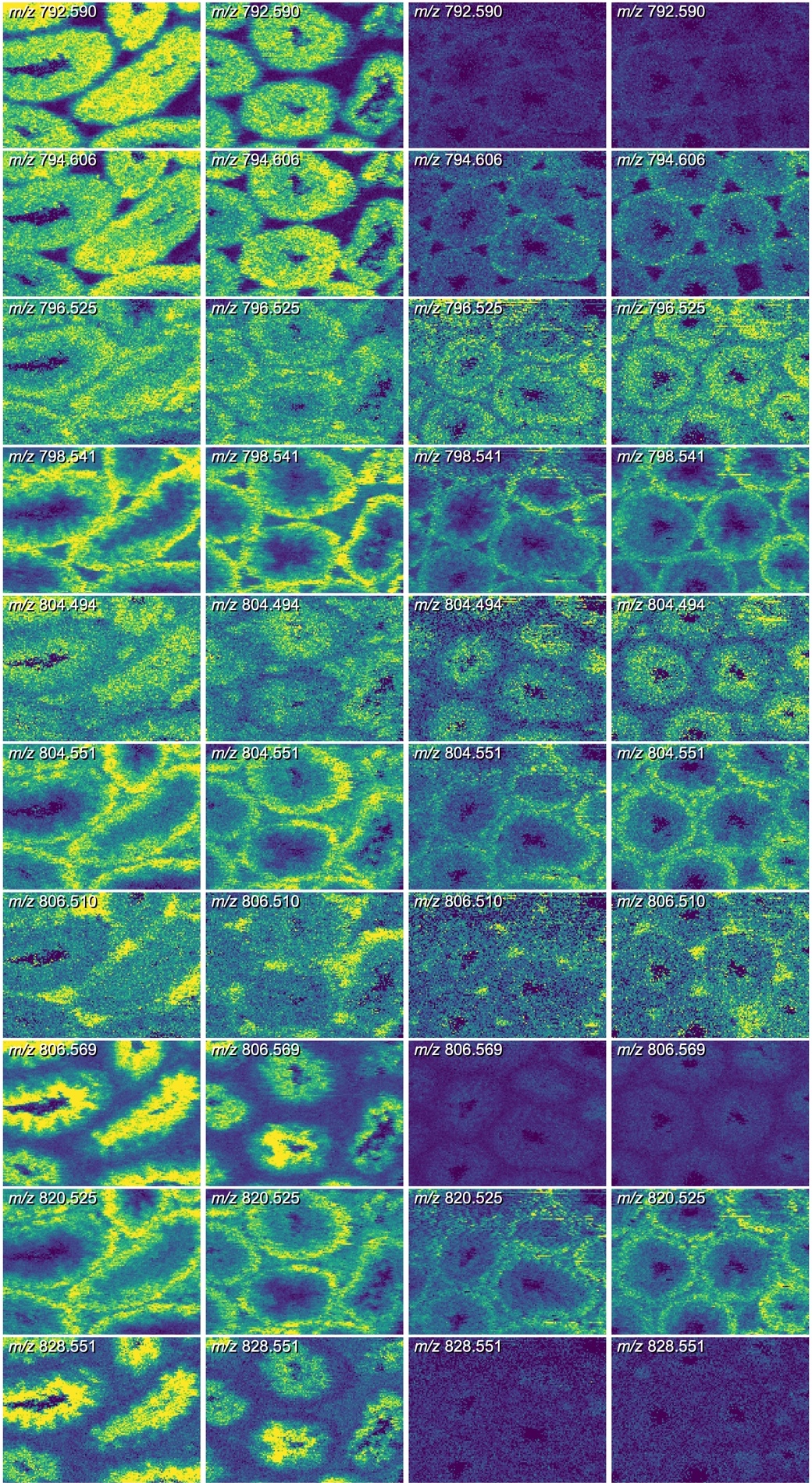


Comparisons of ion images for wild-type (WT) and knockout (KO) testes. The *m/z* values are shown in the images. The two ion images on the left-side show results from WT mice, and the two images
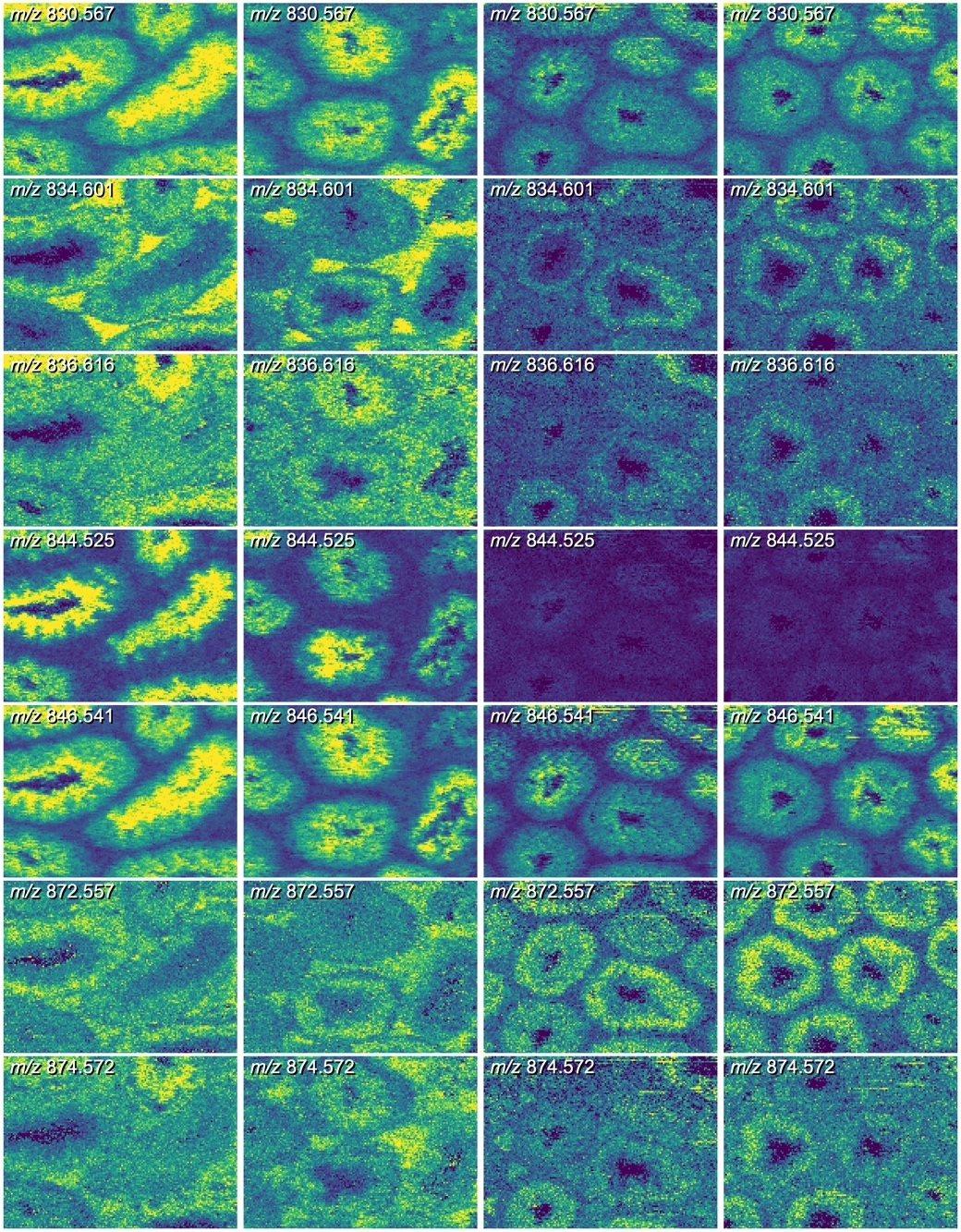
on the right-side show results from KO mice, arranged in the order of increasing *m/z* values. The color scales for the same ions were identical. See Table S2 for assignment details.

# References

1. Iizuka-Hishikawa Y, Hishikawa D, Sasaki J, Takubo K, Goto M, Nagata K, Nakanishi H, Shindou H, Okamura T, Ito C, Toshimori K, Sasaki T, Shimizu T (2017) Lysophosphatidic acid acyltransferase 3 tunes the membrane status of germ cells by incorporating docosahexaenoic acid during spermatogenesis. Journal of Biological Chemistry 292:12065–12076. https://doi.org/10.1074/jbc.M117.791277
